# *Boletim* and *Arquivos*: scientific communication until the creation of the Revista de Saúde Pública

**DOI:** 10.1590/S1518-8787.2016050000115

**Published:** 2016-10-17

**Authors:** Maria Cristina da Costa Marques, Mariana de Carvalho Dolci

**Affiliations:** I Departamento de Prática de Saúde Pública. Faculdade de Saúde Pública. Universidade de São Paulo. São Paulo, SP, Brasil; II Programa de Pós-Graduação em Saúde Pública. Faculdade de Saúde Pública. Universidade de São Paulo. São Paulo, SP, Brasil

**Keywords:** Public Health, Periodicals as Topic, history, Scientific Communication and Diffusion, Review, Historical Article

## Abstract

Based on historical references of scientific communication, we analyzed the issues of the *Boletim do Instituto de Higiene de São Paulo* and of the *Arquivos da Faculdade de Higiene e Saúde Pública da Universidade de São Paulo*. Published respectively from 1919 to 1946 and from 1947 to 1966, they totaled 120 issues. In their 48 years of publication, their goal was to disseminate the scientific production of the institution and to legitimize the theoretical debate of the field, in addition to supporting the public health intervention models, written by leading researchers of the institution and by contributors and managers in the field of public health. Both the *Boletim* and the *Arquivos* were recognized as scientific communication of national reference, and have laid the foundations for the creation of the *Revista de Saúde Pública*, in 1967.

## RESUMO

Com base em referenciais históricos da comunicação científica, foram analisadas as edições do Boletim do Instituto de Higiene de São Paulo e dos Arquivos da Faculdade de Higiene e Saúde Pública da Universidade de São Paulo. Publicadas respectivamente de 1919 a 1946 e de 1947 a 1966, totalizam 120 edições. Em seus 48 anos de publicação, o objetivo era disseminar a produção científica da instituição e legitimar o debate teórico do campo, além de apoiar os modelos de intervenção de saúde pública, escrita por importantes pesquisadores da instituição e colaboradores e gestores do campo da saúde pública. Tanto o Boletim como os Arquivos foram reconhecidos como comunicação científica de referência nacional e lançaram as bases para a criação da Revista de Saúde Pública, em 1967.

## INTRODUCTION

Based on historical references of scientific communication, we analyzed the two periodicals that preceded the *Revista de Saúde Pública*: the *Boletim do Instituto de Higiene de São Paulo* and the *Arquivos da Faculdade de Higiene e Saúde Pública da Universidade de São Paulo*, here designated *Boletim* and *Arquivos*, edited respectively from 1919 to 1946 and from 1947 to 1966. We consulted the digitalized collection available on the Portal de Revista of the Universidade de Sao Paulo (USP) (http://www.revistas.usp.br/bihsp). The 120 issues were organized in database according to topic, authorship, abstract, text type, editors, and other indicators, which allowed us to characterize important reflective contexts of the journals at the time.

This article presents the historical perspective of scientific communication and its main questions to support the central argument: the one that the *Boletim* and the *Arquivos* met the precepts of scientific communication and were a relevant strategy to legitimize the institution to which they were linked and to build a theoretical and practical field – the public health and its affected areas.

### Scientific Communication

Over the centuries, the scientific dissemination have served different interests and motivations. Studies on the topic can elucidate how its variations responded to assumptions of philosophy of science, of scientific contents involved, of cultural codes, of political and economic interests, and of the available means of circulation of ideas in several places and times^15^.

The historiography of scientific communication suggests that the emergence of scientific journals comes from the scientific revolutions that occurred in the 17th century and that were transformed, over time, in primordial functions of preserving the memory of science and spreading scientific knowledge among peers and to the society^13^.

In Brazil, the health scientific communication by journals emerged in the 19th century, with various approaches and sortable in three categories:

- published until 1830, as journals of science, technical arts and literature, varieties, technical works, and translations of foreign articles and without specialized content;
- medical newspapers and journals that published scientific articles and reports of researches or experiments, in addition to translations of foreign scientific works, mostly French, a practice that remained until the end of the 19th century;
- newspapers that published news from the medical area and disclosed epidemics and alerts of public health measures^13^.

In Brazil, the systematic scientific communication was regular and stable only when connected to institutions and when not limited to isolated activities for the dissemination of science. This is noticeable when the first journals of scientific societies founded in the Country emerged, from 1830 on, feature that has remained throughout the 20th century, even considering the new forms of circulation of scientific ideas of the 21st century^10^.

This article is based on the aforementioned assumptions, and assumes that both the creation of the *Boletim*, in 1919, and its replacement by the *Arquivos*, in 1947, observed the characteristics of the scientific communication of alignment to the precepts of science and also to the existing political and academic interests. Over the 48 years of publications and considering their different contexts, one realizes the project of disseminating the scientific production of the institution, as well as of researchers of the area, to legitimize the theoretical debate of the field and support the public health intervention models, especially in the state of Sao Paulo in Brazil. Another important fact, countersigned by the historiography of the topic, is that they were always linked to a scientific institution: the Instituto de Higiene and the Faculdade de Higiene e Saúde Pública of the Universidade de São Paulo.

### Instituto de Higiene

By an agreement signed between the Government of Sao Paulo and the International Health Board, from the Rockefeller Foundation, the Instituto de Higiene succeeded the Laboratório de Higiene, created in 1918 as Chair of the Faculdade de Medicina de São Paulo. It became an autonomous and essential institution, especially in the state of Sao Paulo, in the formulation of health policies, such as the one adopted from 1925 on, whose central axes were health education and health centers^9^.

Geraldo Horácio de Paula Souza and Francisco Borges Vieira were the first scholars indicated by the Rockefeller Foundation for the first course of Hygiene and Public Health, ministered by the newly-opened School of Hygiene and Public Health, from the Johns Hopkins University. Returning to Brazil, Paula Souza took over the direction of the Instituto de Higiene, in 1922, and implemented new methodologies in hygiene and public health. In 1925, no longer associated with the Faculdade de Medicina, the Instituto de Higiene de São Paulo began an independent academic program and secured space in health researches and in the training of personnel in public health, with continued support from the Rockefeller Foundation^9^.

With the financial support from the Rockefeller Foundation and the cession of extensive grounds by the State Government of Sao Paulo, they signed the contract for the construction of the new building, at Avenida Dr. Arnaldo, completed in 1932. The building met the purposes of the academic project of the Instituto de Higiene: it had research laboratories, sections of physiology, sanitary chemistry, occupational hygiene, nutrition and dietetics, microbiology and immunology applied to hygiene, leprology and mycology, sanitary technique, rural hygiene and parasitology, and one small hospital unit. The other areas of the building were designed to educational activities, in addition to the statistical, epidemiology, and social investigations and inquiries sections, as well as to the Centro de Saúde Modelo^9^.

In the early 1940s, the Instituto de Higiene had graduated 49 sanitarians, 466 health educators, and 45 nutritionists and certified other 49 doctors who followed their emergency or intensive courses. On November 29, 1945, in magna session of the Congregation, the Faculdade de Higiene e Saúde Pública of the Universidade de São Paulo was settled.

### *Boletim do Instituto de Higiene de São Paulo* – 1919-1946

The fact of having appeared in the year following the creation of the Laboratório de Higiene da Faculdade de Medicina indicates the importance of the *Boletim* as a vehicle for scientific communication, legitimator and promoter of the ideals of hygienism as founding basis of public health in the period.

In the 28 years in which it was edited, the *Boletim* presented editorial features that help elucidating its role in the legitimization of the institution to which it was linked and also in the consolidation of a basis of understanding and intervention about and on the existing public health.

Each of the 88 edited issues have published one article, offprint, or comment, and its regularity is remarkable – broken only in the years of 1925 and 1926 –, considering the potential difficulties of the processes of creation, structure, and institutional training. The creation of the Laboratório de Higiene in 1918, the change in the management of the Instituto de Higiene in 1922 to Geraldo de Paula Souza, the formalization of the independence of the Instituto regarding the Faculdade de Medicina, the change to the headquarters in 1931, and the installation of the Faculdade de Saúde Pública in November 1945, among other facts, show the dynamism of the institutionalization of the public health field in Sao Paulo and the importance that the historical subjects of the time gave to the scientific communication, instrument that legitimates the desired intellectual identity.

With short intervals between the numbers, the *Boletim* was considered, already in the early years, a publication under the auspices of the State Government of Sao Paulo and of the Rockefeller Foundation, which indicates it as part of the institutional agreement signed between the American foundation, the State Government, and the Instituto de Higiene. Between 1927 and 1935, when the Imprensa Oficial de Estado assumes the editing and publication of the journal, different managers appear, with emphasis on the Escolas Profissionaes do Lyceu Coração de Jesus de Sao Paulo.

Most of the published works are academic productions of directors, researchers, trainers, professors, and assistants attached to the Instituto de Higiene and also – common practice at the time^26^ – offprints published in other periodicals, studies presented at conventions, and lectures given in various occasions.

The first issues of the *Boletim* have published studies, offprints, and conferences of Samuel T. Darling, Wilson G. Smillie, G. H. de Paula Souza, and F. Borges Vieira, head group of the institution in different periods. The topics referred to hygienism and its precepts, prophylaxis of diseases, epidemiological studies and some diseases, feeding, and diseases present in Sao Paulo. The texts of the issues 8 and 9, from 1922, are an exception, respectively with one conference of Emílio Ribas^23^, former director of the Health Service of the state of Sao Paulo, and one of Belizário Penna^22^, from the National Department of Public Health, Director of the Department of Prophylaxis, issued on November 22, 1921, as part of the Rural Hygiene intensive course. The highlight of these two issues presents the renowned sanitarians in the journal, which certainly legitimized it scientifically and politically.

Topics related to health problems in Sao Paulo and to intervention models based on the scientific principles of Hygiene are notorious in several editions. In 1924, the number 20 of the *Boletim* brings an interesting study of Geraldo de Paula Souza and Nicolino Morena entitled “Suggestions for the improvement of the Sanitary State Legislation, on food kinds”^19^ and, in 1928, the issue 29 presents a study of Borges Vieira, “Considerations on the epidemiology of some communicable diseases in the city of Sao Paulo – Brazil”^6^, which shows that the Instituto received epidemiological data of the communicable diseases from the State Health Service, which allowed studies on the incidence and health situation in the capital and in the interior.

At the issue 47 of the *Boletim*, from 1931, with the study of Borges Vieira and Fleury Silveira^8^ “Intestinal protozoa in men at the city of Sao Paulo”, it is possible to understand the structure of the Instituto de Higiene to develop assistance activities and research. The study results from the observation of 373 people served mainly in the Centro de Saúde Modelo, performing the tests in the laboratories of the Instituto. In this line, the models of health services organization and their scientific principles were also topics published in the *Boletim*, with emphasis on the number 59, from 1936, with the text “Main Health Center of Sanitary Organization”, by Geraldo Horacio de Paula Souza and Francisco Borges Vieira^18^.

The institutional partnerships between state organs also appear in several works, for example in number 80, from 1943, with the article by Benjamin Alves Ribeiro^24^, assistant at the Instituto de Higiene de São Paulo, which mentions a cooperation between this, by its Occupational Hygiene Section, and the Instituto de Pesquisas Tecnológicas (IPT), for a project of organization of the medical service in the IPT.

In the penultimate issue of the *Boletim*, from 1945, the report of Borges Vieira discusses a prominent topic in the period: “Health-care issues in the city and field”^7^, article presented at the Brazilian Congress of Post-War Sociomedical Problems, held in Salvador, Bahia, in the same year. The author indicates the need to focus on the “sociomedical” problems exacerbated by post-war conditions for the proposition of sanitary measures. As a channel of communication of studies and scientific and political positions of researchers, managers, and assistants from the Institute, the *Boletim* aligned itself with urgent health discussions on important occasions.

### *Arquivos da Faculdade de Higiene e Saúde Pública da Universidade de São Paulo* (1947-1966)

The *Arquivos* were published for 20 years (1947-1966). In the introduction of the *Arquivos*, Geraldo Horácio de Paula Souza^17^ announced the guiding principles of the new publication:

The *Arquivos da Faculdade de Higiene e Saúde Pública*, which now start their life, will not lose sight of the guidelines drawn by the initiators of this Institution, promoting the publication of the original studies of their researchers and encouraging the collaboration of the best elements of our land (p. 4).

The *Arquivos* gather 194 original articles, published in 20 completed volumes, distributed in 30 fascicles and one supplement. Although without intending to use them exclusively to articles of members of the faculty, their structure caused this to happen, and, guided especially for libraries and scientific societies, their distribution and exchange reduced their penetration in other cores interested in public health^17^.

In 1966, the director of the Faculdade de Higiene e Saúde Pública, Rodolfo dos Santos Mascarenhas, wrote a note in the latest issue of the *Arquivos*, introducing their replacement by the *Revista de Sa*úde Pú*blica*. He informs that the *Arquivos* began in 1947, at the initiative of Geraldo Horácio de Paula Souza, and that they have had biannual publication, under the guidance of the Comissão de Biblioteca of the institution, disseminating exclusively scientific or technical articles drawn up in the institution or with the collaboration of its faculty^14^.

In the first issue, they discussed various topics, with texts by João Alves Meira^16^, Hermelino Herbster Gusmão and Casuhê Yassuda^11^, Raphael de Paula Souza and Hermelino Herbster Gusmão^21^, John Lane^12^, and Dorival da Fonseca Ribeiro and Francisco A. Cardoso^25^.

Unlike the *Boletim*, the *Arquivos* credited in the header of the articles, since the first number, the departments to which the authors belonged and the Professor responsible for the area. From 1953 on, these information were replaced by the qualification of the authors in footnotes.

In the digitalized editions that make up the database consulted in this research, one have access, among other information, to studies presented in national and international conventions, theses, and lectures given on several occasions. The articles discuss hygienism, tuberculosis, entomology, epidemics, chemical studies, intoxications, urban problems, syphilis, nutrition, health surveillance, epidemiology, mortality, among others, held at the Faculdade de Higiene e Saúde Pública and almost all of them related to the state of Sao Paulo in Brazil.

We highlight the issue 2 of volume 4 (December 1950), which brings an article of Elza Salvatori Berquó^5^, from the Department of Statistics, the first woman to publish a text as main author in the *Arquivos*. With the title “About the determination of a moment of any order of a generic centered moment of a sample supposedly coming from a specified normal population at k dimensions”, Berquó presents her associate professor thesis of the Chair of Biostatistics at the Faculdade de Higiene e Saúde Pública of the Universidade de São Paulo, approved in April 1951.

The issue 1 of volume 13 (June 1959) is a commemorative edition of the Jubileu de Prata da Universidade de São Paulo (1934-1959), with article by professor of Civil Law at the Faculdade de Direito of the Universidade de São Paulo, Jorge Americano^1^. The issue 1 of volume 17 honors its founder, Geraldo de Paula Souza (died 1951), and part of issue 2 was dedicated to the memory of professor John Lane (died 1963) and his scientific work.

In 1967, in the editorial of the first issue of the *Revista de Saúde Pública*, the director Raphael de Paula Souza writes that, as occurred in the creation of the *Arquivos*, the appearance of the *Revista de Saúde Pública* did not mean break in continuity. Ideals, principles, and purposes are the same. With its growth, both the Faculdade and *Revista de Saúde Pública* suffer transformations, while remaining inextricably linked to their point of origin – the Laboratório de Higiene da Faculdade de Medicina. And, by observing the same line of action, and under a new form, the *Revista de Saúde Pública* intends to compete more effectively for the advancement of public health in its environment.

## Final Considerations

The scientific communication carried out in the 48 years of publication of the *Boletim* and *Arquivos* was an important strategy of legitimation of the Instituto de Higiene and Faculdade de Higiene e Saúde Pública de São Paulo as producers of scientific knowledge in the several areas of public health. The journals were a significant *locus* of expression of the progressive expansion of new research topics added to the field and expose the complex dimension that public health and its practice field have acquired over the decades.

In this sense, it is legitimate to say that the creation of the *Revista de Saúde Pública* 50 years ago finds solid institutional and scientific support, which, added to other factors, enabled it to achieve respect in the scientific society of the field, and also in the theoretical and practical areas of interest of public health.
